# Pain management after tonsil surgery in children and adults—A national survey related to pain outcome measures from the Swedish Quality Register for tonsil surgery

**DOI:** 10.1371/journal.pone.0298011

**Published:** 2024-03-07

**Authors:** Maria Roskvist, Fredrik Alm, Pia Nerfeldt, Elisabeth Ericsson

**Affiliations:** 1 Ear-, Nose- and Throat Clinic, County Hospital Mälarsjukhuset Eskilstuna, Sweden; 2 School of Health Sciences, Faculty of Medicine and Health, Örebro University, Örebro, Sweden; 3 Department of Otorhinolaryngology, Karolinska University Hospital and Division of Clinical Science, Intervention and Technology, Karolinska Institute, Stockholm, Sweden; University of Minho, PORTUGAL

## Abstract

**Objective:**

The primary aim of this study was to describe the current practice regarding pain management in relation to tonsil surgery among Ear Nose and Throat (ENT) clinics in Sweden. The secondary aim was to determine the impact of the provider’s regime of rescue analgesics on the pain related Patient Reported Outcome Measures (pain-PROMs) from the Swedish Quality Register for Tonsil Surgery (SQTS).

**Materials & methods:**

A descriptive cross-sectional study originating from a validated web-based questionnaire. The survey enrolled one respondent from each ENT clinic (47/48 participated) nationally. Pain-PROMs from the SQTS, recorded from October 2019 to October 2022, were included (8163 tonsil surgeries).

**Results:**

Paracetamol was used by all enrolled ENT clinics as preemptive analgesia. The addition of COX inhibitors was used in 40% of the clinics. Betamethasone was usually administered, to prevent pain and nausea (92%). All clinics gave postdischarge instructions on multimodal analgesia with COX inhibitors and paracetamol. Rescue analgesics were prescribed after tonsillectomy for 77% of adults, 62% of older children, 43% of young children and less often after tonsillotomy. The most frequently prescribed rescue analgesic was clonidine in children (55%) and oxycodone in adults (72%).

A high proportion of patients reported contact with health care services due to postoperative pain (pain-PROMs/ SQTS). Tonsillectomy procedures were associated with the highest rates of contacts (children/adolescents 13–15%; adults 26%), while tonsillotomy were associated with lower rates, (5–7% of children/adolescents). There was no significant difference in the frequency of health care contacts due to pain regarding whether clinics routinely prescribed rescue analgesics or not after tonsillectomy.

**Conclusion:**

The Swedish analgesic regimen after tonsil surgery is good overall. Nevertheless, there is a need for increased awareness and knowledge to achieve optimal patient recovery. Pain-PROM data demonstrate the call for improvement in pain management after tonsil surgery.

## Introduction

### Pain and tonsil surgery

Tonsil surgery is one of the most painful types of surgery in outpatient settings, and pain is a predictable part of the postoperative experience. Inadequate management of pain is common and can have profound implications [1–5]. A significant proportion of patients contact health care services due to inadequate pain control postoperatively [5–8].

Tonsillectomy (TE), total removal of the tonsils, causes moderate to severe pain lasting several days postoperatively and continues to be the predominant method of treating infection-related indications for tonsil surgery. In recent years, partial tonsillectomy/tonsillotomy (TT) has replaced TE in many parts of the world as a treatment for tonsillar hypertrophy in children with obstructive sleep-disordered breathing (OSDB) [9–11]. TT entails subtotal removal of tonsillar tissue, leaving a margin on the tonsillar capsule and surrounding muscles intact, and reducing postoperative pain, the need for analgesics, and the risk of postoperative haemorrhage, which enables faster recovery compared to TE [1,8–12]. However, TT versus TE in adults has been scarcely studied, and few adults undergo TT, even given obstructive indications for tonsil surgery [7,11].

### Postoperative pain management

Inadequate management of postoperative pain increases morbidity with negative psychological changes, prolongs recovery time, decreases quality of life [4,5,13], and increases costs affecting the health care system [14,15]. Management of pain following tonsil surgery needs to encompass efficacy and safety in the immediate perioperative period and address pain following discharge after surgery with both pharmacological and nonpharmacological interventions. Significant demands are placed on children, caregivers and adult patients, as they are required to make accurate assessments and to administer the correct analgesic dosages [1,3,5,16–18]. Pain after tonsil surgery is often poorly managed at home, and studies have highlighted problems with inadequate prescription and administration [3,5,16,17,19]. Along with bleeding and dehydration, pain is a common reason for readmission [6,7,20].

Many studies have been conducted on the subject of optimal postoperative analgesic treatment in relation to tonsil surgery. International consensus has still not been reached, but there is agreement that a multimodal approach, including paracetamol and cyclooxygenase (COX) inhibiting drugs (equal to nonsteroidal anti-inflammatory drugs, NSAIDs), is needed [16,18,21–25].

### Pain management in a Swedish national context

In Sweden, approximately 13000 tonsil operations are performed annually, of which approximately 9000 are performed in children under 18 years of age [7]. In 2013, the Swedish Quality Register for Tonsil Surgery (SQTS), after recording high numbers of unplanned contacts with health care services due to pain after tonsil surgery (26% of children who underwent TE, and 7% after TT), published and implemented national guidelines on the topic, targeting healthy paediatric patients [22]. Adult TE procedures were associated with even higher rates (34%) of unplanned contact [7]. **Adults** were not addressed in the guidelines, although, and there are still no national guidelines that include them today.

The Swedish national guidelines for healthy **children** [22] advocate a multimodal pharmacological approach including administration of paracetamol, clonidine, betamethasone, and a COX inhibitor, pre- or intraoperatively depending on local routines at the clinic. After discharge, pain management at home should be addressed regularly with a combination of COX inhibitors and paracetamol. To healthy children, the guidelines recommend a higher dosage of paracetamol than the package instructions indicate during the first three days postoperatively. These doses have been in the general pediatric postoperative Swedish guidelines [22,26,27] since twenty years, giving a more potent analgesia than regular dosing. The toxic level for a single dose of paracetamol is 150–175 mg/kg orally in healthy children less than 12 years of age, stated by the Swedish Poisons Information Center. The higher recommended dose of paracetamol day 1–3 after surgery is 96 mg/kg/day (24h), max dose/day 1.5 g x 4 followed by a reduced dosage (72 mg/kg/day (24h), max dose 1.0 g x 4) for days 4–8. The guidelines also emphasize the importance of adequate food and drink intake during the treatment. Malnutrition increases the risk of toxicity, due to lower level of glutathione [22,26].

If there is a need for additional pain relief, rescue medication with the alpha-2-adrenergic agonist clonidine (1–2 μg/kg x 3/day (24 h) max dose 150 μg x 3) is recommended, in favour of opioids. This is based on the analgesic effect of clonidine, which does not affect the respiratory drive, a known complication of opioid treatment. Other side effects of opioid treatment, such as nausea and constipation, are also diminished with clonidine, which has a limited effect on gastrointestinal motility [22,23,28]. Opioids should not be prescribed at all for young children with obesity and OSDB [29–33] but can be used in other populations as a last addition to the postoperative multimodal analgesic regimen when needed [18,23,34]. Codeine is strictly advised against for children, in contrast with historical patterns of prescription, due to its clearly documented risk of inducing respiratory depression in hypermetabolizing individuals [21,23,26,33,35,36]. Internationally, codeine and combined preparations with paracetamol are common and partly available without prescription. There are indications, however, that this type of opioid is associated with high risks in children and adults [21,33,37].

The national Swedish guidelines recommend 5–8 days of multimodal analgesic treatment after TE and 3–5 days after TT [22]. Patients are also encouraged to contact health care services in case of insufficient pain management or the need to extend analgesic treatment time. For the national guidelines, a website was launched to aid caregivers and adult patients in administering analgesics, informing them of side effects, and providing recommendations on tapering medication (www.tonsilloperation.se). Based on the national guidelines, recommendations for calculating the correct dose of non-prescription drugs (COX-inhibitors and paracetamol) for healthy children were developed and are accessible via the website [22].

In 2017, Alm et al. [38] published an evaluation of adherence to the national guidelines in a two-year follow-up and found that the implementation process had been mostly successful. Guidelines generally need continuous reinforcement to remain clinical praxis, and it is reasonable to suspect that adherence has diminished over time. Guidelines concerning adults are still missing, and studies indicate that adults and older children suffer from a higher degree of postoperative pain than younger children [1,3,6,7,38]. The present study was conducted to meet the need for reassessment of the current national practice regarding analgesic treatment during and after tonsil surgery at the Ear Nose and Throat (ENT) clinics in Sweden, both in paediatric and adult care, and evaluate the need for new guidelines.

### Objective

The primary aim of this study was to describe the current practice regarding pain management in relation to tonsil surgery among ENT clinics in Sweden.

The secondary aim was to determine the impact of the provider’s analgesic regime of rescue analgesics on the pain related Patient-Reported Outcome Measures (pain-PROMs) from the SQTS.

## Materials and methods

This study was conducted in two sections. The first section was a descriptive cross-sectional analysis based on a questionnaire, and the second consisted of a retrospective cohort study with prospectively collected pain-PROMs from the SQTS.

### The first study section

#### Sample

The directors at all Swedish ENT clinics that provided tonsil surgery, public and private sector (n = 48), were informed of and encouraged to participate in the study. They were asked to suggest a physician in their facility who was regularly involved in tonsil surgery to complete a questionnaire.

An email was sent to all selected physicians, explaining the project, and including a link to access the questionnaire. The questionnaire was completed and submitted online, and following the initial contact, three reminders were sent if needed. The questionnaire was administered in April 14, 2021—May 14, 2021.

#### Design of questionnaire

The questionnaire used in the present study was based on the questionnaire developed and described by Alm et al. (34) in a study regarding pain management of paediatric patients following tonsil surgery. The original questionnaire contained 33 questions [38]and for the present study, additional questions were added to also fit the adult population. The list of questions was sent to a team with high knowledge and experience in tonsil surgery for face and content validation (two senior ENT consultants, one anaesthesiologist, and one registered nurse anaesthetist). The content validity index (CVI) [39] was calculated to evaluate both item content validity (I-CVI) and scale validity (S-CVI/Averaging). The team members individually assessed each question’s relevance using a four-point scale, 1 = not relevant, 2 = somewhat relevant, 3 = quite relevant, and 4 = highly relevant. In addition, they were asked to comment on whether any specific questions should be modified or deleted. I-CVI was calculated as the number of experts giving a rating of either 3 or 4 for each item divided by the total number of experts. S-CVI/Averaging was calculated using the mean of the total I-CVI for the scale. An acceptable value for I-CVI was ≥ 0.78, and ≥0.90 for S-CVI/Averaging as recommended by Polit et al. [39]. The questionnaire initially consisted of 78 questions. Fifteen questions about demographic factors (operative method, day surgery etc.) were excluded from the CVI calculation. Based on feedback from the experts, and low I-CVI, five questions were deleted. In total, the questionnaire finally contained 58 questions with S-CVI/Averaging 0.96; sections concerned analgesic treatment pre, peri- and postoperatively, antiemetics and laxantia, and the national guidelines for children. Intraoperative use of sedative anaesthetic drugs and analgesics (except clonidine) such as propofol, ketamine, morphine and fentanyl were not recorded in this study. The questionnaire sought information about both child and adult patients and included free commentary fields, fixed choice alternatives, dichotomous answers (“yes” or “no”) and four-point Likert scale measurements from “never” to “always”. For final questionnaire, see S1 Appendix.

### The second study section

#### Sample

Data were retrieved from the Swedish Quality Register for Tonsil Surgery (SQTS), which covers approximately 80% of all benign tonsil surgeries in Sweden. In connection with the surgery, the surgeon or clinic administrator registers a perioperative form, including information on age, gender, indication and surgical method. The patient or caregivers are asked to complete a PROM questionnaire regarding postoperative recovery, complications, and symptom relief at 30 days after surgery and again at six months postoperatively (S2 Appendix). In recent years, the response rates for PROM-questionnaires have been approximately 50%.

#### Data collection from the SQTS

The second study section included collection of data on patients registered in the SQTS from October 2019 to October 2022. The focus was pain-PROMs from the 30-day patient questionnaire (S2 Appendix). These were: For how many days after surgery did you take painkillers? How many days after surgery did you start eating regular food? Have you contacted health care services because of pain after the surgery?

The units declaring routine prescription of rescue analgesics were compared with the units without this praxis on the results of the pain-PROM “contacts due to pain”. This variable “contact due to pain” was chosen since it was considered to be less prone to recall bias compared to the other pain-PROMs. The population was divided into age groups (<13 yrs, 13–17 yrs, ≥18 yrs) and tonsillectomy separated from tonsillotomy. Patients from seven clinics were excluded from the analysis due to uncertainty concerning these clinic´s routine on rescue analgesics at different ages. Finally, to evaluate changes in the magnitude of contacts throughout the years, before and after the guideline implementation in 2013, data from January 2012 to November 2022 were analysed concerning the last pain-PROM “contact due to pain”.

### Statistics

Continuous variables are presented as the means, standard deviation (SD) and min/max (range). Categorical variables are presented as numbers (n) and percentages (%). For two-group comparisons of dichotomous variables, Fisher’s exact test was used. Analysis was performed using IBM SPSS Statistics version 28, and all significance tests were two-sided and conducted with a 5% significance level. Commentary fields with free-text answers were analysed qualitatively.

## Results

### The first study section

#### Participating units

A total of 47 of 48 providers (98% response rate) of tonsil surgery nationally participated in the first phase of the study, from the public (n = 32) and private sectors (n = 15). One private clinic did not take part in the survey. Representatives from all participating ENT clinics completed and returned the questionnaire, with varying response rates for some questions. Low response rates were noted concerning questions about perioperative pharmacological treatment, why a complementary questionnaire was sent to a local anaesthesiologist, as recommended by the original representative. The response rate was 74% for the complementary questionnaire.

The respondents’ clinics ranged from small one-surgeon outpatient facilities to university hospitals and differed in service volume. As surgical technique in TE, cold steel dissection dominated in all ENT clinics. In TT, coblation dissection dominated (45%), followed by radio frequency dissection (36%).

All but one ENT clinic performed tonsil surgery on children. The age and weight limits varied, with university hospitals performing surgery on the youngest and unhealthy children (severe OSA, for example). The lower age and weight limits for the participating clinics were on average 2.6 years old (SD 2.3; range: 1–12) and 12.2 kg (SD 2.8; range 5–16), respectively. Sixty-four percent of the clinics performed tonsil surgery in both in- and outpatient settings, 34% only outpatient surgery, and one clinic only inpatient surgery. The average observation time postoperatively before discharge from day surgery was 4.3 (SD 2.0) hours after TE and 3.3 (SD 1.2) hours after TT. The most important contraindications for tonsil surgery in outpatient settings were ASA ≥ 3 and high body mass index. The mean age-related indication for inpatient surgery was 2.7 years old (SD 0.8 range 1–5). Distance from home to the ENT clinic was often a limiting factor for day surgery; some respondents (36%) declared 40–80 kilometres as the limit. Nineteen percent of the ENT clinics had a patient hotel adjacent to the hospital.

#### Pre- and intraoperative pain management

Several strategies were used to manage pain pre- and intraoperatively on the day of surgery. Infiltration of local anaesthetics with or without adrenalin was normally administered in half of the clinics. Topical application via compression with local anaesthetic-soaked gauze with adrenalin was used by 51% in TE and 26% in TT. A corresponding procedure without adrenalin was used by 22% in TE and 12% in TT.

To prevent pain and nausea/vomiting, betamethasone was used by nearly all ENT clinics (92%) intraoperatively at dosages of 2 mg (10–15 kg), 3 mg (15–25 kg), 4 mg (26–50 kg), or 8 mg (> 50 kg). In addition to betamethasone, the most commonly used antiemetic intraoperatively in children and adults was ondansetron (72%).

The most common form of preemptive analgesia was paracetamol, administered to children intravenously (iv) (20 mg/kg) during the intraoperative phase by 68% of the ENT clinics and orally to adults by 68%. In oral administration, a doubled loading dose (2 g) was usually given. COX inhibiting drugs were given intraoperatively to children in 42% of the ENT clinics and to adults in 38%. The most common COX inhibitor was paracoxib (0,5–1 mg/kg) iv to children and adults. Other COX inhibitors (iv) were etoricoxib (1–1,5 mg/kg) (not to children), ibuprofen (4–10 mg/kg), ketolorac (0.3–0.5 mg/kg), and diclofenac (1 mg/kg). Most respondents reported that COX inhibitors were administered just before the end of surgery when abnormal bleeding tendency was excluded. An intraoperative dose of clonidine (iv) was given to healthy children in 77% of the ENT clinics, with dosages ranging from 0,5–4 μg/kg, and to adults in 38%, with dosages ranging from 50–150 μg. Some clinics administered clonidine orally (2 μg/kg) or dexmedetomidine intranasally preoperatively to children for both sedation and preemptive analgesia.

#### Postoperative pain management

All respondents recommended multimodal analgesic treatment, paracetamol combined with COX inhibitors, for children and adults as a basic analgesic regimen (Tables 1 and 2). Instructions on postoperative analgesia were most frequently given at discharge for both children and adults (routine at 83% of the ENT clinics). Approximately half of the respondents (47%) actively advocated around-the-clock administration of analgesics, including at night. A recommendation for tapering analgesic treatment was given to children´s caregivers by 64% and to adults by 53% of the ENT clinics. When pain decreased, analgesics were to be stopped in the following order: opioids, clonidine, paracetamol and finally COX inhibitors, in adherence to guidelines. Routine postoperative follow-up by a nurse via telephone was relatively uncommon (20%).

**Table 1:** Analgesic regime for children after tonsillotomy (TT) and tonsillectomy (TE), routinely used by 47 of 48 ENT clinics in Sweden. Answers expressed in percentages and number of clinics.

	TE	TT
**Regimen**		
Single analgesic regime	0% (0)	0% (0)
Multimodal analgesia (at least paracetamol and COX inhibitor)	100% (47)	100% (47)
**Paracetamol dose**		
24 mg/kg x 4^a^ day 1–3, 18 mg/kg x 4^b^ day 4 -	66% (31)	57% (27)
10–15 mg/kg x 3–4	34% (16)	43% (20)
**COX inhibitor/NSAID dose**		
Ibuprofen 5–7,5 mg/kg x 3–4	100% (47)	100% (47)
**Rescue analgesic routinely prescribed** ^**c**^		
Young children (3–9 yr)	43% (20)	6% (3)
Older children/adolescents (≥10–12 yr)	62% (29)	11% (42)
**Rescue analgesic, most frequently prescribed drug**	TE/TT	
Clonidine (1 μg/kg x 3, max 150 μg x 3)	55% (26)	
Oxycodone (0.15 mg/kg x 3)	34% (16)	
Ketobemidon (0,2 mg/kg x 3–4) (from 20 kg)	2% (1)	
Morfin (0,2 mg/kg x 4)	4% (2)	
Tramadol	0% (0)	
Codeine^d^	0% (0)	

COX = cyclooxygenase inhibiting drugs equal to NSAID = nonsteroidal anti-inflammatory drugs.

In accordance with the guidelines:

^a^ Maximal dose 1,5g x 4 (24h).

^b^ Maximal dose 1g x 4 (24h).

^c^ In the remaining clinics, caregivers or patients must contact health care services (if pain treatment with COX inhibitor and paracetamol is insufficient) before rescue analgesics are prescribed.

^d^ Citodon® consist of codeine and paracetamol.

**Table 2:** Analgesic regime for adults after tonsil surgery, routinely used by 47 of 48 ENT clinics in Sweden. Answers expressed in percentages and number of clinics.

**Regimen**	
Single analgesic regime	0% (0)
Multimodal analgesia (at least paracetamol and COX inhibitor)	100% (47)
**Paracetamol dose**	
1000 mg x 4	89% (42)
1500 mg x 4 day 1–3, 1000 mg x 3–4 day 4-	9% (4)
2000 mg x 4 day 1–3, 1000 mg x 3–4 day 4-	2% (1)
**COX inhibitor/NSAID dose**	
Ibuprofen 400 mg x 3	53% (25)
Naproxen 100 mg x 2	19% (9)
Diclofenak 50 mg x 3	19% (9)
EtoriCOXib 60 mg x 1–2	4% (2)
CeleCOXib 200 mg x 2	4% (2)
**Rescue analgesic routinely prescribed** ^a^	77% (36)
**Rescue analgesic, most frequently prescribed drug**	
Oxycodone (10–20 mg x 2)	72% (34)
Clonidine (38–75 kg:75μg x 3, 76–112 kg: 113 μg x 3, >112 kg: 150 μg x 3)	13% (6)
Morfin 10 mg x 3–4	6% (3)
Codeine^b^ eg Panocode ®, Citodon ® (0,5–1 g x 4)	6% (3)
Tramadol (50 mg x 3–4)	2% (1)
Ketobemidon	0% (0)

COX = cyclooxygenase inhibiting drugs equal to NSAID = nonsteroidal anti-inflammatory drugs.

^a^ In the remaining clinics, caregivers or patients must contact health care services (if pain treatment with COX-inhibitor and paracetamol is insufficient) before rescue analgesics are prescribed.

^b^ Panocode, ®Citodon® consist of codeine and paracetamol.

*Dosage*, *duration and administration forms of postoperative analgesics*. Approximately 60% of the ENT clinics frequently prescribed a higher dose of paracetamol for healthy children, days 1–3 (24 mg/kg x 4), and after three days, the dose was reduced to 18 mg/kg, in accordance with the guidelines (Table 1). This dosage was applied to a larger extent posttonsillectomy (66%) than posttonsillotomy (57%). The remaining ENT clinics recommended paracetamol doses according to package instructions, 10–15 mg/kg x 3–4, although three ENT clinics applied guideline dosages only if the surgeon expected severe pain. For adults, the most frequently recommended dose of paracetamol was 1 g x 4 (89%) (Table 2). The most common first choice of COX inhibitor was ibuprofen: 5–7.5 mg/kg for children, practised by 100%- of the ENT clinics (Table 1) and 400 mg x 3 for adults (51%) (Table 2). There was no difference between the public and private sectors regarding the choice of analgesics.

The recommended number of days of pain treatment after TT was on average 6 days (for children), and after TE on average 9 days for children and 10 days for adults, details presented in Table 3.

**Table 3:** Number of days recommended with pharmacological pain treatment after tonsillotomy (TT) and tonsillectomy (TE), in different age-groups.

	TT	TT	TT	TE	TE	TE	TE
	2–5 yr	6-12yr	3–18 yr	2–5 yr	6-12yr	13–17 yr	≥18 yr
**Days**							
Mean (SD)	5.87 (2.34)	6.11 (2.42)	6.43 (2.52)	8.31 (2.25)	8.91 (2.38)	10.00 (2.52)	9.95 (2.59)
Range	2–14	3–14	3–14	3–14	3–14	3–14	3–14

Most ENT clinics (72%) gave information to caregivers/children regarding alternative administration forms, which varied with age and the individual. The first choice was an oral solution for young children ≤ 6 years of age (92%) and tablets/capsules for older children 7–12 years (51%) and youth 13–18 years (96%). Suppositories were recommended by few ENT clinics (8%) as the primary form of analgesics for young children.

*Rescue analgesics and other adjuvant recommendations*. In addition to paracetamol and COX inhibitors, rescue analgesics were routinely prescribed after TE for adults by 77% of the ENT-clinics, for older children (≥10–12 yr) by 62%, and for young children (≤9 yr) by 43%. Seventeen percent routinely prescribed rescue analgesics to children who underwent TT. In the remaining clinics, caregivers or patients needed to contact health care services if pain treatment with COX inhibitors and paracetamol was insufficient. The most frequently prescribed rescue analgesic for children was clonidine (1 μg/kg x 3) (55% of the ENT-clinics), followed by oxycodone (0.15 mg/kg x 3) (34%) (Table 1). In accordance with the guidelines Codeine was never prescribed to children. Doses of clonidine or oxycodone for children were usually prepared and distributed at discharge from the hospital to cover the first three postoperative days. Two ENT clinics prescribed clonidine as rescue analgesics for children and stated in their written routine that if the caregivers contacted the clinic due to insufficient pain management, adding a prescription of oxycodone for older children was recommended. The most frequent choice of rescue analgesic for adults was oxycodone (72% of the ENT clinics) and three (6%) used Panocod ® (paracetamol + codeine) (Table 2).

In the free-text responses, many respondents highlighted difficulties in the administration of clonidine to children. Each ENT clinic must implement a routine for administration since the drug is normally not handled by the pharmacy. This was the reason why some ENT clinics did not use clonidine according to the national guidelines. Clonidine was regarded as safe and efficient by the vast majority of respondents. A few thought it to be insufficient, causing sedation and/or risk in patients with cardiovascular disease. Many respondents pointed out the complexity of opioid prescription, stating that on one hand it is an efficient and potent analgesic, but on the other hand, it is associated with a risk of respiratory depression (in younger children with OSDB), nausea and constipation as well as addiction/abuse.

The ENT clinics did not normally prescribe antiemetics after tonsil surgery (89%), but 6% recommended over-the-counter antiemetics. Prescribing laxantia was also uncommon, but 13% recommended this in cases of concomitant opioid prescription. Discharge instructions on physical and dietary restrictions were given after tonsil surgery, with variations in duration and contents between clinics.

#### Opinions on the national guidelines

The majority of respondents stated that there is a need for national guidelines on postoperative analgesic treatment for both children and adults. Most respondents also regarded the national Swedish guidelines for children as safe and comprehensive. A few pointed out difficulty in communicating with child patient caregivers about the need for higher dosages of paracetamol on days 1–3. Most respondents (77%) had taken part of the website, www.tonsilloperation.se, and 68% used it for preoperative and postoperative information. The dose calculator for children was used by 40% in the postanaesthetic care unit (PACU) and 49% recommended it as additional information for caregivers. Most ENT clinics (85%) also handed out their own printed patient information pamphlet.

Several respondents (40%) stated a need for further education on analgesic management in relation to tonsil surgery and 30% answered that they did not know if there was a need.

### The second study section

#### Patient-reported pain-related outcome measures

Between October 2019 and October 2022, ENT clinics participating in this national mapping reported 18,464 tonsil surgeries to the SQTS. The 30-day postoperative questionnaire was returned by 44% (n = 8163) of the caregivers/patients, and their pain-PROMs are presented in Table 4. A high proportion of patients reported postoperative contact with health care services due to pain, particularly in adolescents (15%) and adults (26%) who had undergone TE.

**Table 4:** Patient-reported outcome measures concerning pain (pain-PROMs) from the Swedish Quality Register for Tonsil Surgery, including data from the clinics participating in national mapping. Data period: October 2019 and October 2022.

	Total	TT	TT	TE	TE	TE
		0–12 yr	13-17yr	0–12 yr	13–17 yr	≥18 yr
	N = 8085	N = 3303	N = 104	N = 1245	N = 654	N = 2779
**Days with analgesics**						
Mean (SD)	8.5 (4.8)	5.8 (3.5)	8.0 (4.6)	8.3 (3.9)	10.2 (4.4)	11.3 (4.9)
Missing	576	292	12	98	34	140
**Days to regular food**						
Mean (SD)	5.6 (4.7)	3.3 (3.3)	4.4 (4.2)	5.3 (4.4)	7.0 (4.6)	8.1 (4.9)
Missing	231	116	5	52	64	46
**Contact due to pain**						
% (N) Missing	15.1 (1207) 95	6.8 (223) 32	4.8 (5) 2	12.9 (158) 18	15.0 (280) 11	25.5 (700) 32

TT = Tonsillotomy TE = Tonsillectomy.

The ENT clinics that routinely prescribed rescue analgesics for the TE group had no significant difference in the proportion of patient-reported contacts with health care services compared to clinics with no rescue analgesic routine. Caregivers/children from the clinics that did not routinely prescribe rescue analgesics after TE, contacted health care services due to pain in 13% of cases in the age group <13 years, and 20% in the age group 13–17 years. This can be compared to children/caregivers from ENT clinics that routinely prescribed rescue analgesics to TE, where 13% in the group <13 years, and 19% in group 13–17 years contacted healthcare due to pain (<13 years group comparison: p = 0.726 and 13–17 years group comparison: p = 0.916). Adults (≥ 18 years) from ENT clinics that did not routinely prescribe rescue analgesics contacted health care services due to pain in 27% versus 25% in clinics that routinely prescribed rescue analgesics (p = 0.211).

**Fig 1:**
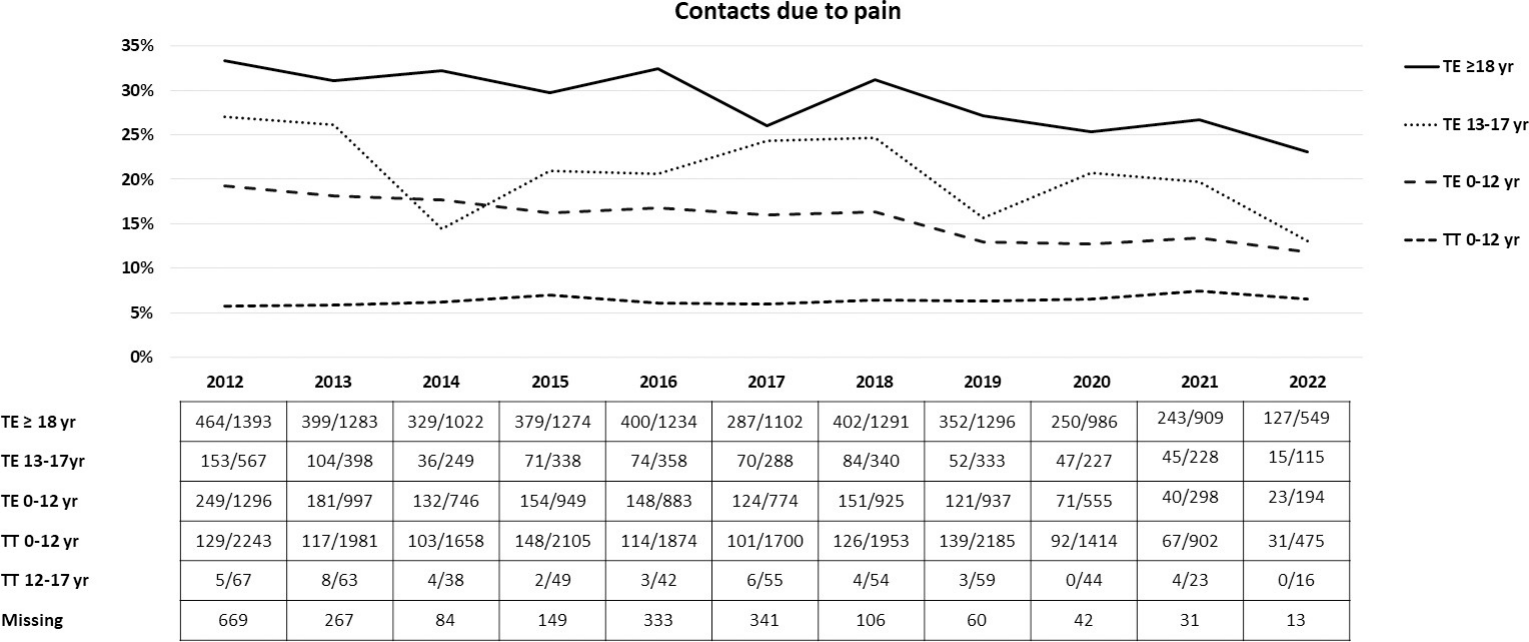
The proportion of reported contacts with health care services due to pain among patients operated on from January 2012 –November 2022 by the clinics participating in the present national mapping. *The number of patients in the tonsillotomy 12–17 yr group was small and therefore not presented in the figure*. TE = Tonsillectomy TT = Tonsillotomy.

The proportion of patients reporting contact with health care due to pain after TE has dropped post-implementation of the guidelines in 2013 (Fig 1). In total 27% of the TE patients contacted health care services due to pain in 2012, compared to 19% in 2022.

## Discussion

### Overall reassuring results

This study evaluated pain treatment practices in relation to tonsil surgery in all but one ENT clinic in Sweden.

The national analgesic regimen after tonsil surgery was found to be good overall, the clinics clearly make an effort to provide the most effective postoperative pain management upon discharge. All ENT clinics recommended a multimodal analgesic regimen postoperatively with both COX inhibitors and paracetamol for children and adults after TE and TT, and the majority of them also routinely prescribed additional rescue analgesics after TE for older children and adults. No clinic prescribed codeine and only a few used paracetamol combined with codeine to adults, none to children. This is reassuring since these drugs are advised against in the context.

### Concerns regarding SQTS data

Pain-PROMs from the SQTS 30 days questionnaire still indicate high numbers of health care contacts due to pain. However, these results need to be interpreted with caution. This questionnaire cannot adequately evaluate the postoperative pain experience as it is limited to three short questions and do not address, for example, the severity of pain the first week after surgery, if the pain subsides with treatment, the presence of side effects or other complicating factors (e.g., inadequate information). This might be part of the explanation for why the present data could not illustrate an impact of rescue analgesics on the pain-PROMs from the SQTS. The ENT clinics that routinely prescribed rescue analgesics limited to only the first three days and encouraged the patients to contact the hospital again if needed, also affects the figures making it seem like a higher number needed to contact health care services due to excessive pain. The same is true if patients reported contact with health care services when receiving a routine follow-up phone call from a nurse. The national website urges caregivers and adult patients to contact the provider in case of insufficient effect of the recommended analgesic regime. An SQTS study showed that caregivers contacted health care services due to pain more frequently if they had studied the patient information on the website compared to caregivers who did not visit the website [6] The PROMs have been questioned in previous studies [6,7], and since November 2022 they have been revised by the SQTS as to better capture the satisfaction rate and patient’s/caregiver´s concerns with the postoperative analgesic treatment given.

### Adherence to guidelines

Evidence to guide safe and efficient management of postoperative pain after tonsil surgery is increasing with numerous advancements in pain treatment standards [21]. There seems to be a trend of decreasing levels of patients contacting health care services due to excessive pain, as shown by data from the present study. This supports that Swedish postoperative pain management of tonsil surgery has improved and that the implementation of the Swedish national guidelines for children has been successful. Adherence has increased, now seven years after implementation, compared to the results previously presented after two years [38]. In the present survey 62% of the ENT clinics routinely prescribed rescue analgesics for older children compared to 50% earlier and 66% prescribed higher doses of paracetamol in the first three days compared to 30% earlier. The process of implementing the guidelines was rigorous, with publications in several journals and multidisciplinary education days held for physicians and nurses who specialized in ENT and anaesthesia. Specific feedback reports from the SQTS allow clinics to compare their own results with others, which has also been helpful in the process. Nevertheless, time passes, and education needs to be repeated, as mentioned by several respondents.

### Choice of drugs

#### Preventive and perioperative analgesia

In this study, a gap was seen in the awareness of the surgeons concerning preventive analgesia, which is why those questions were instead answered by anaesthesiologists. There are several multimodal strategies covering the preoperative period that can help decrease postoperative pain, and preventive analgesia starts before incision or surgery [40], underscoring the importance of the multidisciplinary team setting.

Half of the ENT-clinics in the survey (50%) routinely administered perioperative local infiltration in the tonsillar bed. A meta-analysis has shown a modest effect on pain by this measure but also serious side effects [41]. The national guidelines recommend local anaesthetic-soaked gauze applied to the wound surface instead [22], and these were also used to a great extent. Most anaesthesiologists administered betamethasone routinely and added ondansetron intraoperatively. This is in accordance with the guidelines for the prevention of postoperative nausea and vomiting [22], with betamethasone also reducing pain in the early postoperative period [42]. Many children suffer from nausea and constipation postoperatively after discharge, naturally contributing to a worse general condition.

#### Postoperative analgesia

There is still controversy internationally regarding which postoperative analgesics are most efficient in diminishing pain following tonsil surgery, although a combination is clearly needed. Timing, dosage, route of administration, form and duration are of utmost importance. This requires careful counselling of caregivers who are in charge of their children’s recovery after surgery [2,16,19,23,43]. It is important that providers counsel patients and caregivers repeatedly to anticipate, assess, reassess, and correctly treat postoperative pain, which is highlighted by national and international guidelines [18,21,22,25,44].

A multimodal approach, including COX-inhibitors and paracetamol and if necessary, clonidine or oxycodone, to healthy children and adults, seems to be the most effective way to manage postoperative pain following discharge. To reach acceptable pain levels and improve recovery, routine prescription of rescue analgesics needs to be implemented on a wider scale. Most respondents saw a satisfying effect of clonidine as a rescue analgesic in children, and the remaining ENT clinics can use this as a reference. There are no studies on the effectiveness and safety of clonidine in home settings after tonsil surgery, but there is a large bank of national clinical experience regarding the use of clonidine in hospitals [22,26,27,45]. The majority of adults were prescribed rescue analgesics (oxycodone), but a high proportion still reported contact with health care services due to excessive pain.

The risk of respiratory depression must always be accounted for when opioids are needed postoperatively, especially in OSAS patients as well as in young and obese children [21,33,44,46]. Most Swedish ENT-clinics preferred clonidine to children and adolescents because it has no effect on respiratory drive, the tendency of bradycardia is rare and the most common side effect, sedation, is dose dependent. Further studies focused on the effect of clonidine in addition to adequate baseline analgesia (paracetamol and COX-inhibitor) following tonsil surgery are needed [21,44].

No respondent prescribed tramadol to children or adolescents and only one provider prescribed it to adults, which is in line with the Swedish guidelines, due to its risk of confusion and respiratory depression [17], as well as nausea, dizziness, constipation, headache and intoxication [22,26,46,47]. None of the ENT clinics prescribed codeine for children, which is gratifying since this has been a serious problem in the past [21,30,33]. There are indications that this type of opioid is also associated with high risks in the adult population [37], and hopefully, the few providers who still prescribe codeine will reconsider.

One can speculate that adult patients might need more detailed information on the postoperative analgesic regimen. There might also be a gain in prescribing paracetamol and COX-inhibitors for this group instead of recommending over-the-counter medications for clarity in dosages.

### Duration of analgesic treatment

Previous studies of pain at home following tonsil surgery report that pharmacological pain management may be needed for up to three weeks postoperatively after TE and more than one week after TT [4,5,48,49]. To meet the patient’s individual needs, instructions from professionals regarding pain treatment should be flexible in terms of treatment length. Some might need a longer time of treatment with analgesics after tonsil surgery, which can be supported by data from the present study, at least concerning older children and adults.

### Informing patients and/or caregivers

Many providers found use of the website www.tonsilloperation.se as an aid in both prescribing medications and providing information to patients. ENT clinics could possibly achieve more sufficient pain management for their patients by routinely handing out written information on postoperative pain management after discharge. Timing of this information is also important; it is more beneficial to discuss postoperative analgesics at the doctor’s office in conjunction with deciding on an operation than postoperatively when the patient might already be affected by pain [2,43]. It is crucial to explain the rationale for analgesic selection, dosage and around-the-clock regime including during night, along with potential side effects, to children’s caregivers and adult patients. Confusion can be caused by the fact that the automated calculation tool on the website presents caregivers with dosages according to guidelines, while 40% of the ENT clinics do not recommend dosage according to it. Concordant and distinct information will facilitate reaching compliance with the guideline´s slightly complicated dosage, resulting in a less painful situation for the patient.

### Limitations

The greatest limitation of this study was reliance on questionnaire data without the ability to clarify. Additionally, the data were collected from only one physician at each ENT clinic, although they were asked to answer for their group of colleagues in general. The physicians may have been selected due to their knowledge on for example guidelines for pain treatment in tonsil surgery. Individual preferences among surgeons in each clinic concerning the choice of analgesic regimes certainly occur to a lesser or greater extent. However, 47 of 48 ENT clinics in the nation participated, giving good representation. Another important limitation is that the national mapping of the first study section was cross-sectional, while the data from the SQTS in section two were prospectively collected and covered a much longer period, giving a mismatch in study design.

## Conclusion

The Swedish national analgesic regimen after tonsil surgery with a multimodal approach is good overall, and it is clear that ENT clinics make an effort to provide optimal pain relief. This is the result of a rigorous guideline implementation process leading to gradual change in the direction of optimal analgesic treatment in the paediatric population. Nevertheless, there is a need for an increase in awareness and knowledge to achieve optimal patient recovery and pain-PROM data demonstrate a call for improvement in pain management after tonsil surgery. Further studies are needed, especially among adult patients, for whom there might be a need to develop national guidelines.

## Supporting information

S1 Appendix QUESTIONNAIRE: Pain management after tonsil surgery in children and adults—a national survey related to pain outcome measures from the Swedish Quality Register for Tonsil Surgery. (DOCX)

S2 Appendix A description of the structure and data collection in the Swedish Quality Register for Tonsil Surgery during 2009–2022. (DOCX)
