# Supplementary material for: Pain management after tonsil surgery in children and adults—A national survey related to pain outcome measures from the Swedish Quality Register for tonsil surgery
Source: PLoS One. 2024 Mar 7;19(3):e0298011. doi: 10.1371/journal.pone.0298011 (PMC10919603; doi:10.1371/journal.pone.0298011)
Supplement: S1 Appendix — (DOCX) [file pone.0298011.s001.docx]

| **S1 Appendix** **QUESTIONNAIRE** **Pain management after tonsil surgery in children and adults - a national survey related to pain outcome measures from the Swedish Quality Register for Tonsil Surgery** | |
| --- | --- |
| **AO=ANSWER OPTIONS** | |
| **QUESTIONS DEMOGRAPHIC FACTORS** | |
| **1&2** | ***Which surgical technique (s) are used for tonsillectomy (1) tonsillotomy (2) at your clinic on children and adults?*** |
|  | a) Cold steel dissection b) Radio frequency dissection ex Coblation ( < 0.5MHz) c) Radio frequency dissection ex Ellman-Surgiton, Sutter-Curtis (>4 MHz) d) Diathermic scissors d) Ultracision e) Bipolar diathermy f) other named technology_____ |
|  | AO: “usually”, “sometimes”, “seldom”, “never” |
| **3** | ***At our clinic, we perform (3a) tonsillectomy and (3b) tonsillotomy on children*** |
|  | From age (years)____ From weight (kg)__ |
| **4** | ***At our clinic, we perform tonsil surgery on (4a) children and (4b) adults as*** |
|  | AO: “only inpatient surgery” “only outpatient surgery” “inpatient and outpatient surgery” |
| **5** | ***What age limit and/or weight limit does your clinic have to perform tonsil surgery in outpatient settings on children*** |
|  | From age (years)____ From weight (kg)__ |
| **6** | ***Which criteria apply to when tonsil surgery should be performed in inpatient care for (6a) children (6b) adults?*** |
|  | Enter: ASA-classification, Age, BMI-limit, Presence of hematological infection, Distance between home address and operating clinic (Swedish miles), Other factors of concern |
| **7** | ***In case of outpatient surgery, how many hours of observation time is recommended before discharge after tonsillectomy for children and adult patients?*** |
|  | Enter number of hours + free commentary field |
| **8** | ***In case of outpatient surgery, how many hours of observation time is recommended before discharge after tonsillotomy for children and adult patients?*** |
|  | Enter number of hours + free commentary field |
| **9** | ***Who is responsible for discharging the patient when the operation is performed as planned day surgery for children and/or adult patients?*** |
|  | Open answers option |
| **10** | ***Does your clinic utilize a patient hotel in connection with tonsil surgery for children and adult patients?*** |
|  | AO: “Yes” “No” |
| **11** | ***If yes: Which criteria apply to using the patient hotel for patients undergoing tonsil surgery for children and adult patients?*** |
|  | Open answers option |
| **12** | ***If yes: What does the procedure for discharge from the patient hotel look like? Who is responsible for discharge?*** |
|  | Open answers option |
| **13** | ***Are there locally designed written guidelines (PM) at your clinic for pain management at home after tonsil surgery for (13a) children (13b) adults?*** |
|  | AO: “Yes” “No” |
| **14** | ***Are there individual variations regarding choice of pain management among the ENT surgeons at your clinic? Feel free to comment!*** |
|  | AO: “No”, To a small extent”, “To a large extent”, “Don´t know” + Free commentary field |
| **15** | ***Who has the primary responsibility for prescribing postoperative pain treatment at home after the tonsil surgery for children and adult patients?*** |
|  | Open answers option |

| **GENERAL QUESTIONS ABOUT PAIN MANAGEMENT** | |
| --- | --- |
| **1** | ***When is the most common time to prescribe analgesics for postoperative pain management? Enter an option. (1a) to caregivers/child (1b) to adults*** |
|  | AO: ”In the outpatient clinic in conjunction with operative planning”, ”Preoperatively on the day of surgery” ,“Postoperatively at discharge” |
| **2** | ***At your clinic, do you normally prescribe analgesics or recommend them over the counter after (2a) child tonsillotomy (2b) child tonsillectomy (2d) adult tonsillotomy (2e) adult tonsillectomy?*** |
|  | AO: ”Prescription”, ”Over the counter” |
| **3** | ***Which modalities are used at you clinic for giving information on analgesics post-tonsil surgery to (3a) caregivers/children (3b) adults?*** |
|  | AO: “Written material”, “Oral councelation”, ”Both written and oral information” + free commentary field |
| **4** | ***If written information is given to children/caregivers, which material do you use?*** |
|  | ***(4a) Pre-printed info sheets written by the clinic, (4b) Printed information from the website tonsilloperation.se, (4c) Oral recommendation to visit the website tonsilloperation.se & print info*** |
|  | AO:” Never”,” Sometimes”, ”Always”, ”Don’t know” and free commentary field |
| **5** | ***Have you read the info on the website tonsilloperation.se?*** |
|  | AO: “Yes”, “I am aware of the website, but have not taken part of it”, “No, I´ve never heard of it” |
| **6** | ***Answer the following statements. How do you normally use the website tonsilloperation.se at your clinic?***  ***(6a) As preoperative information (6b) As postoperative information (6c) We use the interactive dosage calculator as information to caregivers (6d) We use the interactive dosage calculator as a working tool (Crib for prescription)*** |
|  | AO:” Never”, ” Sometimes”, ”Always”, ”Don’t know” |
| **7.** | ***Does your clinic apply routine follow up telephone wise postoperatively by a nurse to caregivers and/or adult patients?*** |
|  | AO: “Yes”, “No”, ”Don’t know”, If yes: On which postoperative day does the nurse call? |
| **8.** | ***In case of follow-up according to question nr 7, does the information from these phone calls reach you as a doctor?*** |
|  | AO: “Yes”, “No” & Please give examples in the free commentary field |
| **9&10** | ***Do you give restrictions concerning food intake to children/caregivers and adult patients after (9) tonsillotomy (10) tonsillectomy?*** |
|  | AO: “Yes” , “No” , If yes: Please clarify the restrictions and duration. |
| **11** | ***Do you give restrictions concerning physical activity to children/caregivers and adult patients after tonsil surgery?*** |
|  | AO: “Yes”, “No”, If yes: Please clarify the restrictions and duration. |
| **QUESTIONS ON ANALGESIC DRUGS, DOSAGE AND ADMINISTRATION FORM FOLLOW:** | |
| ***Pre- or intraoperative analgesia to children and adults. Do you use…:*** | |
| **12** | ***Betametason (Betapred®) (pre or intraoperatively) to (12a) children (12b) adults?*** |
|  | Enter dose (mg/kg), usual route of administration (orally or intravenously) and when (pre- or intraoperatively). (Plus alternative ”not used”) |
| **13A** | ***Paracetamol (pre and/or intraoperatively to (13Aa) children, (13Ab) adults?*** |
|  | Enter dose (mg/kg), usual route of administration (orally or intravenously) and when (pre- or intraoperatively). |
| **13B** | Route of administration: Grade 1-3 with 1 as the commonest. Enter separate grading for children and adults. AO: “po”, ”pr”, “IV” and free commentary field |
| **13C** | ***Paracetamol (in the postoperative unit) to (13Ca) children, (13Cb) adults*** |
|  | Route of administration: Grade 1-3 with 1 as the commonest. Enter separate grading for children and adults. AO: “po”, ”pr”, “IV” and free commentary field |
| **14** | ***Klonidin (pre and/or intraoperatively) to (14a) children, (14b) adults*** |
|  | Enter dose (mikrograms/kg), usual route of administration (orally or intravenously) and when (pre- or intraoperatively). |

| **15** | ***COX inhibitor/NSAIDs (pre and/or intraoperatively) to (15a) children, (15b) adults*** |
| --- | --- |
|  | Enter dose (mg/kg), usual route of administration (orally or intravenously), when (pre- or intraoperatively) and which NSAID is most commonly used. |
| **16** | ***COX inhibitor/NSAIDs eg Celecoxib (eg Celebra®) and Parcecoxib (eg Dynastat) (pre and/or intraoperatively) to (16a) children, (16b) adults*** |
|  | Enter dose (mg/kg), usual route of administration (orally or intraveinously) and when (pre- or intraoperatively). |
| **17** | ***Ketorolak/Toradol® (pre and/or intraoperatively) to (17a) children, (17b) adults*** |
|  | Enter dose (mg/kg), usual route of administration (orally or intravenously) and when (pre- or intraoperatively). |
| **18** | ***Other analgesic drugs (eg opioids) and/or complementary comments on pre- and intraoperative analgesia to children and adult patients*** Open answers option |
| **19&20** | ***Do you use local anesthetics to children and adult patients during tonsillectomy (19) tonsillotomy (20)?*** |
|  | a) Compression gauze **with** local anesthetic with adrenalin b) Compression gauze **without** adrenalin, c) Infiltrative anesthetic **with** adrenalin d) Infiltrative anesthetic **without** adrenalin |
|  | AO: “Always”, “Sometimes” Seldom” “Never” ”Don’t know” |
|  | **ANTIEMETICS AND LAXANTIA** |
| **21** | ***Which antiemetic drug do you normally use in tonsil surgery to children and adult patients? Please comment and give examples, e.g. drug combinations.*** |
|  | AO: Ondansetron (Zofran®), Droperidol (Dridol®), Prometazin (Lergigan®), Postafen, Primperan, Don´t know, Free commentary field |
| **22** | ***Do you, as a routine, prescribe antiemetics to children and adult patients after tonsil surgery?*** |
|  | AO: “Yes”, “No”, “No, but we recommend antiemetics over the counter”, If yes: Which antiemetic is most commonly used? |
| **23** | ***Do you normally prescribe or recommend laxantia to children and adult patients after tonsil surgery?*** |
|  | AO: “Yes Always”, ”Yes, but only when prescribing opioids”, ”No”, ”If yes: Which laxantia do you normally use?” Free commentary field |
| **POSTOPERATIVE ANALGESICS TO CHILDREN** | |
| **24A** | ***Do you recommend COX inhibitors/NSAIDs to children <18 years old after tonsil surgery at your clinic?*** |
|  | AO: “Yes”, “No, NSAIDs are not recommended,” If yes: Which NSAID is normally recommended to children < 18 years old? Free commentary field |
| **24B** | ***What dosage do you prescribe at your clinic of COX inhibitors/NSAIDs (Ibuprofen and Diklofenac)?*** |
|  | a) Ibuprofen: Enter mg/kg x times per day, b) Diklofenac: Enter mg/kg x times per day, c) Other NSAID: Name and enter mg/kg x times per day. Any comments on NSAIDs as analgesic treatment in tonsil surgery? Free commentary field |
| **24C** | ***Which form of COX inhibitor/NSAIDs is usually recommended to children of the following years of age: < 4, 4-6, 7-12 , 13-18?*** |
|  | AO:” Tablet/Capsule”, “Oral suspension”, “Suppository” |
| **25** | ***Is COX-2 inhibiting drugs such as Celecoxib (eg.Celebra®) and Parcecoxib (eg. Dynastat®) used for children at your clinic?*** |
|  | AO: “Yes”, “No”, ”Don´t know” ,If yes: On which indication and how often? Free commentary field |
| **26A** | ***Which is the prescribed maximal dose of paracetamol (po/pr) for children postoperatively? Enter mg/kg x times a day Free commentary field*** |
|  | Which dose do you prescribe days 1-3 postoperatively? Days 4-10? Free commentary field |
| **26B** | ***Which route of administration form of paracetamol do you normally recommend to children <18 years of age?*** |
|  | AO: ”Orally”, ”Rectally”, Feel free to comment! Free commentary field |
| **26C** | ***Which form of paracetamol is usually recommended to children < 4, 4-6, 7-12, 13-18 years of age?*** |
|  | AO: “Coated tablet “, “Mouth-dissolving tablet“, “Effervescent tablet“, “Oral solution“, “Suppository” |
| **27** | ***Do you give information on alternate forms and routs of administration of analgesics to caregivers/children as a routine?*** |
|  | AO: “Yes”, “No”, Please comment on your reasoning and information regarding this! Free commentary field |
| **28** | ***Do you prescribe the same postoperative analgesia to children …..*** |
|  | *…after tonsillectomy and tonsillotomy?* |
|  | AO: “Yes”, “No”, If not: What is the difference? Free commentary field |
|  | *…regardless of age of the child/youth?* |
|  | AO: “Yes”, “No”, ” If not: What is the difference? Free commentary field |
| **29** | ***Do you prescribe additional analgesics (rescue) to complement NSAIDs and paracetamol (eg Clonidine or Oxicodone) after tonsil surgery to…?*** |
|  | (29a) younger children posttonsillotomy? (29b) younger children posttonsillectomy? (29c) older children/youths posttonsillotomy? (29d) older children posttonsillectomy? |
|  | AO: “Always”, “No, caregivers need to contact our clinic if NSAIDs and paracetamol is insufficient” |
| **30** | ***If you prescribe rescue analgesics to children, which drug is normally used? If more than one alternative is used, please motivate why!*** |
|  | AO: ”Clonidine/Catapresan”, ”Oxikodon/Oxynorm”, ”Ketogan”, ”Morfin”, ”Tramadol”  Enter comments on choice of drug, administration and/or motivate why several drugs are used.  Free commentary field |
| **31A** | **The choice of rescue analgesics to children CLONIDINE** |
|  | ***Clonidine is used by a number of ENT-clinics in Sweden. Which advantages do you see in using this drug in conjunction with tonsil surgery?*** Open AO |
| **31B** | ***Clonidine is used by a number of ENT-clinics in Sweden. Which disadvantages do you see in using this drug in conjunction with tonsil surgery?*** Open AO |
| **32A** | **The choice of rescue analgesics to children OXICODONE** |
|  | ***Oxicodone is used by a number of ENT-clinics in Sweden. Which advantages do you see in using this drug in conjunction with tonsil surgery?*** Open AO |
| **32B** | ***Oxicodone is used by a number of ENT-clinics in Sweden. Which disadvantages do you see in using this drug in conjunction with tonsil surgery?*** Open AO |
| **33A** | **The choice of rescue analgesics to children, KETOGAN** |
|  | ***Ketogan is used by a number of ENT-clinics in Sweden. Which advantages do you see in using this drug in conjunction with tonsil surgery?*** Open AO |
| **33B** | ***Ketogan is used by a number of ENT-clinics in Sweden. Which disadvantages do you see in using this drug in conjunction with tonsil surgery?*** Open AO |
| **34A** | **The choice of rescue analgesics to children MORPHINE** |
|  | ***Morphine is used by a number of ENT-clinics in Sweden. Which advantages do you see in using this drug in conjunction with tonsil surgery?*** Open AO |
| **34B** | ***Morphine is used by a number of ENT-clinics in Sweden. Which disadvantages do you see in using this drug in conjunction with tonsil surgery?*** Open AO |
| **35** | ***Do you use combined preparations with codeine eg Citodon or Tramadol as postoperative analgesia after tonsil surgery to children at your clinic?*** |
|  | AO for both drugs: “Yes”, “No”, ”Don´t know” Please comment! Free commentary field |
| **36A** | ***Do you give information to caregivers on tapering of analgesia including in which order the drugs are to be stopped when pain decreases?*** AO: “Yes”, “No” |
| **36B** | ***If yes to question 36A: What does the tapering regime look like? Enter in which order the named drugs are to be stopped. Feel free to comment!*** Open AO |
| **37** | ***Do you actively recommend administration also during nighttime to children at your clinic?*** |
|  | AO: “Yes”, “No”,” Don´t know”, Feel free to comment! Free commentary field |
| **38-40** | ***How many days of analgesic treatment do you recommend to children after tonsil surgery at your clinic…?*** ***Answer for age group: 2-5, 6--12, 13-18 years of age*** |
|  | Enter number of days for each age group after tonsillotomy and tonsillectomy. |
| **POSTOPERATIVE ANALGESIA TO ADULTS** | |
| **41** | ***Paracetamol as postoperative analgesia to adults:***  ***What maximal dose and number of administration occasions per day do you recommend to adults after tonsil surgery?*** |
|  | Enter maximal dose/kg/day and number of administration occasions |
| **42** | ***COX inhibitors/NSAIDs as postoperative analgesia to adults:*** |
|  | Enter which NSAID is most often recommended, maximal dose/kg/day and number of administration occasions |
| **43** | ***Do you use Cox2-inhibitors eg Celocoxib (eg Celebra®) and Parcecoxib (eg Dynastat®) to adults at your clinic?*** |
|  | AO: “Yes”, “No”, “Sometimes”, ” Don’t know” If yes: On which indication and how often? Free commentary field |
| **44** | ***Do you use CODEINE/combined preparations eg Citodon to adults as postoperative analgesia after tonsil surgery at your clinic?*** |
|  | AO: “Yes”, “No”, “Sometimes”, ”Don´t Know” Please comment your answer! Free commentary field |
| **45** | ***How many days of analgesics do you generally recommend to adults after tonsil surgery?*** |
|  | Enter number of days and free commentary field |
| **46** | ***Do you routinely prescribe rescue analgesics (Clonidine, Oxicodon, Morphine, Ketogan) as a complement to NSAIDs + paracetamol after tonsil surgery?*** |
|  | AO: “Yes”, “No, the adult patient needs to contact the clinic if NSAIDs and paracetamol is insufficient” |
| **47** | ***If yes: Which rescue analgesic is usually prescribed to the adult patient?*** |
|  | AO: ”Clonidine/Catapresan®”, ”Oxicodone (Oxynorm®)”, ”Ketogan”, ”Tramadol” ,”Codeine/combined preparations eg Citodon®”, ”Other Opioid name in the commentary field”, “No rescue analgesic is prescribed routinely”. Free commentary field: Name opioid and/or motivate why if several analgesics are used. Feel free to comment on the need for rescue analgesics post tonsil surgery! |
| **50** | ***Do adults receive information, as a routine, on tapering of analgesics? In that case, in which order are the drugs to be stopped?*** Open answers option |
| **QUESTIONS CONCERNING BOTH CHILDREN AND ADULTS** | |
| **51** | ***Which unit do you recommend to children/caregivers and adult patients to contact after tonsil surgery in case of insufficient pain control?*** Open answers option |
| **52** | ***Do you see a need for further education concerning analgesics in relation to tonsil surgery?*** |
|  | AO: “Yes”, “No”, ”Don´t know” Free commentary field: Do you have suggestions of content in such education? |
| **53** | ***I/we think there is a need for national recommendations on pharmacological treatment of pain in relation to tonsil surgery for (53a) children (53b) adults*** |
|  | AO: “Completely agree”, “Somewhat agree”, “Somewhat disagree”, “Completely disagree” |
| **54** | ***Do you apply the national guidelines for pharmacological treatment of pain and nausea in relation to tonsil surgery at your clinic?*** |
|  | AO: ”Yes”, ”Yes with exceptions (Specify any exceptions in the free commentary field)”, ”No (If no: Specify your reasoning in the free commentary field.)”, ”I do not know about the guidelines” |
| **55** | ***To which extent do you estimate that your clinic is adherent to the national guidelines for children in relation to tonsil surgery?*** |
|  | AO: ”Low”,”Medium”,”High” |
| **56** | ***To which extent do you agree with the following statements after the guidelines for children where implemented?***  *”The guidelines are patient safe,” The guidelines are clear”, ” The guidelines provide optimal pharmacological treatment to children”, ”Children and caregivers easily understand the prescriptions”, ”Children and caregivers are compliant to the ordinations”, ”The postoperative well-being of the children has improved” , ”Children have less pain”, ”Children present lower incidence of nausea”,” Children present lower incidence of constipation, ”Children return to normal food intake quicker”* |
|  | AO: “Completely agree”, Somewhat agree”, Somewhat disagree”, “Completely disagree” |
| **57** | ***Do you, at your clinic, find any difficulties or disadvantages in following the guidelines for children?*** |
|  | AO: “Yes”, “No”, If yes: Please comment! Free commentary field |
| **58** | ***Do you see any change in the general adherence to the guidelines for children over time, during recent years?*** |
|  | AO: “Yes”, “No”, ”Don’t know “, “No”, If yes: In which way? Free commentary field |
