# Supplementary material for: Pain management after tonsil surgery in children and adults—A national survey related to pain outcome measures from the Swedish Quality Register for tonsil surgery
Source: PLoS One. 2024 Mar 7;19(3):e0298011. doi: 10.1371/journal.pone.0298011 (PMC10919603; doi:10.1371/journal.pone.0298011)
Supplement: S2 Appendix — (DOCX) [file pone.0298011.s002.docx]

| **S2 Appendix.** **A description of the structure and data collection in the Swedish Quality Register for Tonsil Surgery during 2009-2022.** A detailed description is available at <https://ton.registercentrum.se/inenglish/the-national-tonsil-surgery-register/p/HJV8b8hV>. |
| --- |
| **First questionnaire (day of surgery).** |
| • Date of surgery |
| • Social security number (age, date of birth, gender) |
| • Outpatient- or inpatient surgery |
| • Indication: *upper airway obstruction/snoring/ tonsil hypertrophy, chronic tonsillitis, recurrent tonsillitis, peritonsillitis, systemic complications to tonsillitis, or “other”- namely...* |
| • Surgical method: *TE- tonsillectomy, TEA- tonsillectomy with adenoidectomy, TT- tonsillotomy, TTA- tonsillotomy with adenoidectomy* |
| • Surgical technique for dissection: *cold steel, diathermy scissors, ultracision, laser, dissection with bipolar diathermy, radiofrequency, or “other technique”- namely...* |
| • Surgical technique for haemostasis (in addition to compression): *infiltration of local anaesthetics with epinephrine, unipolar diathermy, bipolar diathermy, ligation, suture ligation, radiofrequency, or “other” technique- namely...* |
| • Postoperative bleeding that occurred during hospital stay and required intervention: *Yes/No.* If yes, what type of intervention? *Tranexamic acid/Desmopressin, Return to theatre, Blood transfusion, Other.* |
| **Second questionnaire. PROM 30 days after surgery** |
| • Name, social security number, date |
| • Have you contacted medical care due to bleeding from the throat? *(Yes/ No)* |
| • If yes, how many days after surgery did the bleeding occur? |
| • Have you been admitted to hospital due to bleeding from the throat? *(Yes/ No)* |
| • If yes, which hospital where you admitted to? |
| • Was another surgery performed due to bleeding? |
| • Did any infection occur during hospital stay or within 30 days of surgery? *(Yes/ No).* If yes, what kind of infection? |
| • Have you contacted medical care due to the infection? |
| • Have you received antibiotics due to the infection? |
| • Have you contacted medical care because of pain after the surgery? *(Yes/ No)* |
| • For how many days after surgery did you take painkillers? |
| • How many days after surgery did you start eating regular food? |
| • Was the information you received in accordance with your experience of the surgery and the period after the surgery? • If no, what wasn ́t? |
| • Have you read the patient information at [www.tonsilloperation.se](http://www.tonsilloperation.se) |
| **Third questionnaire. PROM 6 months after surgery.** |
| Name, social security number, date |
| “Check the alternative that best describes your/ your child’s situation”: *The problems are gone, My problems are almost gone, My problems remain, My problems have worsened* |
| Have you (your child) had some other complaints? (Yes/No). If yes, namely...? |
